# On-Site Inactivation for Disinfection of Antibiotic-Resistant Bacteria in Hospital Effluent by UV and UV-LED

**DOI:** 10.3390/antibiotics13080711

**Published:** 2024-07-29

**Authors:** Takashi Azuma, Masaru Usui, Tomohiro Hasei, Tetsuya Hayashi

**Affiliations:** 1 Department of Pharmacy, Osaka Medical and Pharmaceutical University, Takatsuki 569-1094, Japan; tomohiro.hasei@ompu.ac.jp (T.H.); tetsuhaya456@gmail.com (T.H.); 2 Food Microbiology and Food Safety, Department of Health and Environmental Sciences, School of Veterinary Medicine, Rakuno Gakuen University, Ebetsu 069-8501, Japan; usuima@rakuno.ac.jp

**Keywords:** hospital wastewater, ultraviolet-light emitting diode (UV-LED), UV lamp, disinfection, antimicrobial-resistant bacteria (AMRB), antimicrobial resistance genes (AMRGs), wastewater treatment plant (WWTP), river environment

## Abstract

The problem of antimicrobial resistance (AMR) is not limited to the medical field but is also becoming prevalent on a global scale in the environmental field. Environmental water pollution caused by the discharge of wastewater into aquatic environments has caused concern in the context of the sustainable development of modern society. However, there have been few studies focused on the treatment of hospital wastewater, and the potential consequences of this remain unknown. This study evaluated the efficacy of the inactivation of antimicrobial-resistant bacteria (AMRB) and antimicrobial resistance genes (AMRGs) in model wastewater treatment plant (WWTP) wastewater and hospital effluent based on direct ultraviolet (UV) light irradiation provided by a conventional mercury lamp with a peak wavelength of 254 nm and an ultraviolet light-emitting diode (UV-LED) with a peak emission of 280 nm under test conditions in which the irradiance of both was adjusted to the same intensity. The overall results indicated that both UV- and UV-LED-mediated disinfection effectively inactivated the AMRB in both wastewater types (>99.9% after 1–3 min of UV and 3 min of UV-LED treatment). Additionally, AMRGs were also removed (0.2–1.4 log_10_ for UV 254 nm and 0.1–1.3 log_10_ for UV 280 nm), and notably, there was no statistically significant decrease (*p* < 0.05) in the AMRGs between the UV and UV-LED treatments. The results of this study highlight the importance of utilizing a local inactivation treatment directly for wastewater generated by a hospital prior to its flow into a WWTP as sewage. Although additional disinfection treatment at the WWTP is likely necessary to remove the entire quantity of AMRB and AMRGs, the present study contributes to a significant reduction in the loads of WWTP and urgent prevention of the spread of infectious diseases, thus alleviating the potential threat to the environment and human health risks associated with AMR problems.

## 1. Introduction

The emergence and spread of antimicrobial-resistant bacteria (AMRB) is becoming an increasingly worldwide problem [1,2,3]. The importance of considering not only the environmental health risks and effects of AMRB that are transmitted via humans and animals but also the indirect risks posed to the environment has gained increasing focus [4,5]. The O’Neill Commission that was commissioned by the UK government estimates that if effective action is not taken to address the spread of AMRB, annual global deaths will rise from 0.7 million in 2014 to 10 million by 2050, which is more deaths than are caused by cancer, and the economic loss to global GDP is estimated to be $100 trillion [6]. These projections are currently becoming a real threat, as recent reports have indicated that the number of AMRB-related deaths has almost doubled to 1.27 million as of 2019 [7]. In Japan, an action plan focused on antimicrobial resistance (AMR) has been implemented since 2016 to reduce AMRB prevalence [8,9], and a detailed assessment of the current situation and revision of the action plan to promote further action is underway.

Effluents from hospitals and other healthcare facilities contain a wide variety of microorganisms originating from patients [10,11,12]. Antimicrobial use in Japan is approximately 15.8 defined daily doses (DDD) per 1000 adults [13], and this tends to be lower than that of the rest of the world (an average of approximately 20 DDD in Europe and reportedly over 60 DDD in India [14]). In contrast, a worldwide survey of clinical practice reported that drug resistance rates for microorganisms exhibit a typical antimicrobial resistance trend, including penicillin-resistant *Streptococcus pneumoniae* (PRSP) at 48%, methicillin-resistant *Staphylococcus aureus* (MRSA) at 51%, carbapenem-resistant *Pseudomonas aeruginosa* (CRPA) at 17%, and third-generation cephalosporin-resistant *Escherichia coli* (CREC) at 18% [4,15,16,17]. Among these, Japan possesses one of the highest rates of resistance in the world to PRSP and MRSA, whereas CRPA and CREC are also at high levels [18]. Therefore, understanding the actual AMRB in medical wastewater, assessing the risk to the environment, and developing technologies that can reduce or eliminate such risks can contribute not only to protecting human health but also to reducing nosocomial infections and improving the quality of healthcare [19,20,21]. Additionally, the AMRB issue is also important in regard to ensuring the safety of the water environment and the protection of watersheds on a large scale, and it is also considered an important issue in the context of balancing sustainable human prosperity and global environmental protection [2,22,23].

Several methods are known to inactivate microorganisms in wastewater, including chlorination, Fenton, ozone, and photocatalytic oxidation [24,25,26]. All of these methods are known to be effective in inactivating microorganisms and have been studied for adaptation to hospital wastewater treatment methods [21,27]. However, they require the addition of chemicals for treatment, and the residual chemicals after treatment are often an issue [28,29]. On the other hand, disinfection with ultraviolet light (UV) is effective for inactivating microorganisms without the addition of chemicals that are resistant to chlorine disinfection such as *Cryptosporidium* and other microorganisms [30,31]. UV disinfection primarily uses UV lamps that emit low-wavelength (254 nm) UV light that is considered to be highly effective in inactivating microorganisms. However, UV lamps use mercury discharge to generate ultraviolet light, and therefore, the environmental impact of mercury use cannot be ignored. Additionally, the voltage required to drive the lamps is high, maintenance costs are high due to the short lamp life, and there is not much flexibility in the design of the treatment equipment [32,33]. Recently, ultraviolet light-emitting diodes (UV-LEDs) have undergone rapid technological development in recent years and possess many excellent advantages such as mercury-free functionality, low voltage requirements, and a 10-fold longer life span than UV lamps. Currently, their future use as a replacement for UV lamps is becoming a reality [32,34,35].

The low-wavelength UV light that can be emitted by UV-LEDs currently on the market is at 255, 265, 280, 300, and 365 nm. However, it has been reported that UV irradiation doses at 255 nm and 265 nm are extremely low compared to UV lamps using existing technology, and problems remain due to equipment limitations when considering implementation and application in a stand-alone disinfection process [36,37,38]. Additionally, ultraviolet rays above 300 nm are the same as long-wave ultraviolet rays (>290 nm) [39] that are close to the visible light contained in sunlight reaching the surface of the Earth and are not suitable for use as an inactivation treatment for large volumes of water such as wastewater in terms of disinfection effectiveness [40,41]. Therefore, when considering alternatives to 254 nm UV lamps that are known to be effective in regard to inactivating microorganisms, evaluations have primarily focused on a new UV wavelength of 280 nm that is believed to be capable of providing high-power UV irradiation at lower wavelengths [34,42]. To date, research has led to the evaluation of inactivation effects and the development of methods for a wide variety of environmental contaminants such as dyes, pesticides, and pharmaceuticals [32,43,44] and also for common indicator microbes such as *E. coli* and *Enterococci* [45,46,47], viruses [30], fungi [38], and COVID-19 [48,49]. However, only a limited number of studies have attempted to evaluate the inactivation of AMRB in wastewater using UV-LED.

There is a worldwide lack of knowledge on the study of hospital wastewater, and few reports on the actual status of environmental pollutants and their impact on the environment, as well as on treatment [50,51,52]. The treatment of hospital wastewater at the source with high concentrations is more effective in reducing the impact of environmental pollutants on the environment than treating large volumes of diluted wastewater at WWTPs, as is the case with many other types of wastewater [53,54,55]. Therefore, evaluating the effectiveness and efficiency of disinfection treatments using UV lamps and UV-LED to inactivate AMRB in hospital wastewater is expected to provide a more comprehensive understanding of the emerging water pollution problem caused by AMRB and yield valuable insights for predicting and assessing the environmental risks associated with the release of AMRB into the environment.

In this study, to evaluate the effectiveness of UV lamps and UV-LED in the context of disinfection treatment of pharmaceutical bacteria in wastewater, treatment tests were first conducted using wastewater obtained from a wastewater treatment plant (WWTP) and wastewater obtained from a hospital as wastewater from a medical facility, and the inactivation effect was evaluated. Next, based on the results obtained, the effectiveness of UV-LED as a new disinfection treatment method for environmental risk reduction by comparing the effectiveness of AMRB inactivation by UV and UV-LED and by assessing the effectiveness of using UV-LED on medical wastewater was evaluated.

## 2. Materials and Methods

### 2.1. Sampling

Wastewater samples were collected from a WWTP and a hospital located in an urban area of Japan as described previously [56]. The WWTP treats municipal sewage generated by a population of 420,000 individuals. The WWTP influent was first treated with conventional activated sludge and discharged as the WWTP secondary effluent. The WWTP secondary effluent was treated with chlorine (1.2 mg NaClO/L for 15 min) for disinfection and then discharged into the river as WWTP effluent. Hospital effluent was collected directly from a hospital with 480 beds and an average of 1200 patients per day. Hospital effluent is discharged into municipal sewage, merged with domestic and industrial wastewater, and eventually introduced into the WWTP influent. Samples were collected in December 2022 on the days when the influence of precipitation was low and when the recorded rainfall was >1 mm for the preceding two days [57]. Basic water quality parameters of WWTP wastewater and hospital effluent were pH 7.5 and 8.4, 19 mg/L and 51 mg/L for chemical oxygen demand (COD), 5.2 mg/L and 74 mg/L for biochemical oxygen demand (BOD), 12 mg/L and 204 mg/L for suspended solids (SS), 2.4 mg/L and 30 mg/L for NH_4_-N, 1.0 mg/L and 2.3 mg/L for PO_4_^3−^-P, 167 CFU/mL and 2600 CFU/mL for *E. coli*, and 617 CFU/mL and 108,000 CFU/mL for coliform bacteria, respectively. A stainless-steel pail sampler was used to collect the wastewater samples, which were then placed into separate sterilized glass bottles. Sodium thiosulfate (0.5 g/L) was immediately added to each bottle to quench the residual chlorine [58,59]. All samples were immediately transported to the laboratory in a cooler box (within 2 h), stored at 4 °C in the dark, and processed within 12 h.

### 2.2. Disinfection of Wastewater by UV and UV-LED

Inactivation testing of AMRB and antimicrobial-susceptible bacteria (AMSB) using UV and UV-LED was based on previous reports of inactivation of microorganisms by the UV-LED system [45,60,61] and also on various guidelines for the light attenuation of environmental contaminants [62,63,64]. The hospital effluent was dispensed into a sterilized glass Petri dish with a diameter of 120 mm and an effective volume of 220 mL and maintained in the dark at 20 °C, as this is the annual mean water temperature of WWTPs in Japan [65]. UV irradiation was performed using a low-pressure mercury lamp (3UV-38; Funakoshi Co. Ltd., Tokyo, Japan) at a peak wavelength of 254 nm. UV-LED irradiation was supplied by a UV-LED system (PearlBeam, NIKKISO Co., Ltd., Tokyo, Japan) with peak emission at 280 nm. The UV irradiation intensity (mW/cm^2^) was measured within the UV region (240–480 nm) using a UV radiometer (UVPadE; Opsytec Dr. Gröbel GmbH, Ettlingen, Germany). UV irradiation was performed by irradiating the dish with UV light from a UV or UV-LED source that was attached to the top of the dishes [34,47]. The UV and UV-LED irradiation systems are presented in Figure S1. UV and UV-LED irradiation intensities were fixed by adjusting the distance between the irradiator and the dish to ensure that the same irradiation intensity (0.3 mW/cm^2^ for UV and 0.3 mW/cm^2^ for UV-LED, which is approximately the maximum intensity value of UV-LED irradiation intensity) was used in the inactivation experiments. Samples were collected after 0, 0.2, 0.3, 0.5, 1, and 3 minutes of exposure.

In a similar previous study, no significant differences (*p* < 0.05) were observed for proliferation or attenuation for both AMRB and AMSB at 7 h at 20 °C under dark conditions [66]. As the inactivation treatment time used in this study was much shorter, the present investigation directly expressed the inactivation effect of microorganisms based on the results obtained by UV irradiation. The duration of inactivation was determined based on the average treatment conditions (3.6 ± 1.8 min and 277 ± 164 J/m^2^ as UV fluences) in WWTPs that use UV disinfection in Japan [65] and on the values reported in previous studies [38,45,60]. In this study, the UV fluence (mJ/cm^2^) was calculated by multiplying the UV irradiation intensity (mW/cm^2^) by the inactivation treatment time (min) and the transmittance of UV light in the water samples [67,68]. Finally, the solutions were analyzed using chromogenic agar methods as described in Section 2.3.

### 2.3. Microbial Analysis

Six representative classes of AMRB have been assigned a global priority by the WHO [69,70] and addressed in the clinical area [5,71,72] and five classes of antimicrobial-susceptible bacteria (AMSB) used as the microbial species comprising each ARB that was targeted were also investigated as reported previously [66,73]. The lists and abbreviations of the target AMRB and AMSB are presented in Table 1.

The prevalence of each type of AMRB and AMSB in the wastewater samples was determined by screening individual microbes grown on chromogenic agar formulations. ChromID CARBA was used for the detection of CRE, chromID ESBL was used for the detection of ESBL-E, chromID MRSA was used for the detection of MRSA, ChromID VRE New was used for the detection of VRE (bioMérieux S.A., Marcy-l’Étoile, France), CHROMagar MDRA was used for the detection of MDRA, and CHROMagar MDRP was used for the detection of MDRP (Kanto Chemical Co., Inc., Tokyo, Japan). Similarly, the amount of AMSB was estimated by screening for individual microbes grown on different chromogenic agar formulations devoid of antimicrobials. CHROMagar Acinetobacter was used for the detection of *Acinetobacter*, CHROMagar *Pseudomonas* was used for the detection of *Pseudomonas aeruginosa* (*P. aeruginosa*) (Kanto Chemical Co., Inc., Tokyo, Japan), chromID S. aureus Elite was used for the detection of *Staphylococcus aureus* (*S. aureus*), chromID CPS Elite was used for the detection of *Enterococcus* (bioMérieux S.A., Marcy-l’Étoile, France), and XM-G agar was used for the detection of *E. coli* (Nissui Pharmaceutical Co., Ltd., Tokyo, Japan). AMRB and AMSB were detected following the protocols provided by the manufacturers of the growth media using methods [74] that were described previously [75,76,77,78,79,80]. Ultrapure Milli-Q water (18.2 MΩ·cm; MilliporeSigma, Watford, UK) with a pH adjusted to 7.0 and a 10 M sterilized phosphate buffer were used for dilution.

An aliquot (1 mL) of the water sample was poured onto each agar plate and quickly spread over the surface. The wastewater was aspirated several times with a pipette prior to collection and mixed well before the treated water sample was collected for measurement. Each plate was then covered with a cover plate and incubated at 42 ± 1 °C for 24 h for CRE and ESBL-E and at 37 ± 1 °C for 24 h for the other bacteria in the dark. The bacterial species were differentiated based on the color and morphology of the colony in accordance with the manufacturer’s specifications and as described previously [74,81,82]. The experiments were conducted in triplicate for each case. Colonies were counted, and the number of bacteria that were recovered was expressed as colony-forming units per milliliter (CFU/mL). This was then converted into mean yearly values. If the mean CFU was a whole number, the values were expressed as the nearest integer after application of the rounding-off rule and were counted as N.D. (not detected) if the values were <1. The relative reproducibility values (n = 3) for the AMRB (CRE, ESBL, MDRA, MDRP, MRSA, and VRE) and AMSB (*Acinetobacter*, *Enterococcus*, *E. coli*, *P. Aeruginosa*, and *S. aureus*) were 13%, 9%, 10%, 19%, 11%, and 13% and 14%, 12%, 16%, 9%, and 19%, respectively.

### 2.4. Quantitative PCR (qPCR) Analysis

Quantification of *β*-lactam resistance genes (*bla*_IMP_, *bla*_TEM_, and *bla*_CTX-M_) that are frequently encountered in clinical sites for CRE (*bla*_IMP_) and ESBL (*bla*_TEM_ and *bla*_CTX-M_) as representative ARGs was performed using Quantitative PCR (qPCR) analysis as described previously [11,56,83]. Briefly, 50 mL aliquots of wastewater samples were concentrated using a membrane filter (0.45-μm pore size, Millipore Sigma, Burlington, MA, USA), and genomic DNA was extracted using a DNeasy^®^ Blood & Tissue Kit (QIAGEN, Hilden, Germany) [84,85,86]. Sample pretreatment was conducted using TB Green Premix Ex Taq II (Tli RNaseH Plus; Takara Bio Inc., Shiga, Japan) in 20 μL reaction mixtures containing 3 μL of the DNA template and 0.4 μM of each primer (Table S1) [87,88,89,90]. qPCR was performed using a LightCycler^®^ 480 System II instrument and its respective software (Roche Diagnostics, Basel, Switzerland). Reaction conditions were as follows: initial denaturation at 95 °C (30 s) was followed by annealing for 45 cycles at 95 °C (3 s each) and elongation at appropriate temperatures (57–62 °C) for 30 s. The copy numbers of the bacterial 16S rRNA genes were also determined from the same extracts to normalize values across samples and compare the bacterial abundance between each wastewater sample [91,92,93]. The primers and standard curves that were used to validate the amplification efficiency and linearity (*r*^2^) of each gene are summarized in Table S1. The concentrations of the plasmid stock solutions extracted to make the standard curve were 4.5 × 10^10^, 3.2 × 10^10^, 3.1 × 10^10^, and 3.2 × 10^10^ copies/μL for *bla*_IMP_, *bla*_TEM_, *bla*_CTX-M_, and 16S rRNA, respectively. A standard curve was prepared for the plasmid stock solution by making a 10-fold staircase dilution and using it for qPCR. Sample concentrations were within the range of the standard curve. Analyses were conducted in duplicate, and the mean and standard error were calculated.

### 2.5. Bacterial Community Structure Analysis

Bacterial community structure analysis was performed using MiSeq platform analysis as described previously [94,95,96]. Genomic DNA was extracted from the water samples using an Extrap Soil DNA Kit Plus v.2 (Nippon Steel Eco-Tech Corporation, Tokyo, Japan), and DNA concentrations and purifications were assessed using a Qubit ^®^ 3.0 fluorometer (Thermo Fisher Scientific, Waltham, MA, USA) and a Qubit^®^ dsDNA BR Assay Kit (Thermo Fisher Scientific, Waltham, MA, USA) [97,98]. The V1–V2 region of the bacterial 16S ribosomal RNA (rRNA) gene was used to characterize the bacterial communities [99,100], and the universal bacterial primers 27F/338R were used for PCR amplification [101,102]. PCR was performed using a T100 Thermal Cycler (Bio-Rad Laboratories, Hercules, CA, USA). Finally, the amplified genes were sequenced on a MiSeq platform (Illumina Inc., San Diego, CA, USA) according to the manufacturer’s instructions and previous reports [103,104,105]. Sequence data were pre-processed and analyzed using the Flora Genesis software v.20161108 (Repertoire Genesis Inc., Osaka, Japan). Operational taxonomic units (OTUs) were selected using the open-reference method at a 97% identity level and annotated from the prefiltered Greengenes Database v.13.8 by the UCLUST algorithm [106,107], and taxonomy was assigned using the Ribosomal Database Project classifier at a confidence threshold of 0.80 [108,109].

### 2.6. Statistical Analysis

The data for the tested traits were analyzed using Microsoft Excel software (Office 2019) and are presented as mean values with their individual standard deviation values. A paired *t*-test was conducted to evaluate the difference in inactivation rates between water samples with a statistical significance of *p* < 0.05 [110,111].

## 3. Results and Discussion

### 3.1. Disinfection of the WWTP Wastewater and Hospital Effluent by UV and UV-LED Treatment

The current situation regarding the occurrence of AMRB in the WWTP (influent, secondary effluent, and effluent) and hospital effluent is presented in Table 2. All AMRB targeted in this study were detected in all wastewater samples. The concentrations of AMRB and AMSB ranged from 35 CFU/mL to 963 CFU/mL and 50 CFU/mL to 20,000 CFU/mL in the WWTP influent, from N.D. to 17 CFU/mL and N.D. to 664 CFU/mL in the secondary effluent, from N.D. to 17 CFU/mL and 1 CFU/mL to 140 CFU/mL in the WWTP effluent, and from 31 to 215 CFU/mL and 63 CFU/mL to 13,000 CFU/mL in the hospital effluent, respectively. These results revealed that AMRB was widely present in the wastewater and could be mostly removed during the wastewater treatment process at the WWTP, although some of them were discharged into the river as effluent. These concentrations are typically within a range similar to those described in previous reports on antimicrobials in hospital wastewater in Japan [66].

**Figure 1:**
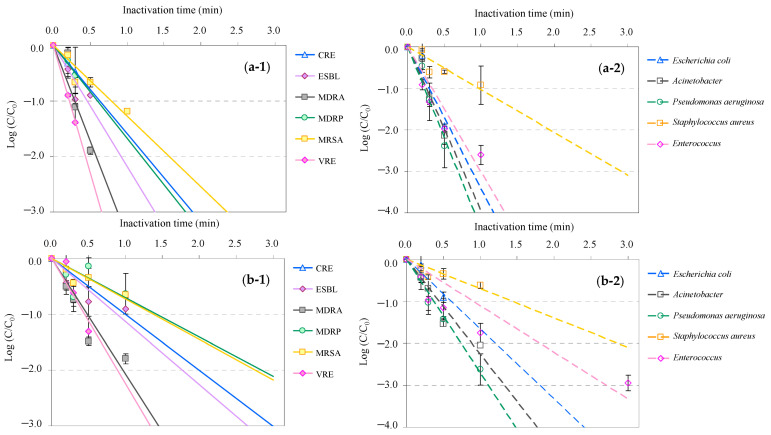
Relative antimicrobial-resistant bacteria (AMRB) and antimicrobial-susceptible bacteria (AMSB) under UV and UV-LED treatment of WWTP wastewater: (**a-1**) UV for A MRB, (**a-2**) UV for AMSB, (**b-1**) UV-LED for AMRB, and (**b-2**) UV-LED for AMSB (C_0_, initial bacterial count; C, bacterial count after treatment; CRE, carbapenem-resistant *Enterobacterales*; ESBL-E, extended-spectrum *β*-lactamase-producing *Enterobacterales*; MDRA, multi-drug-resistant *Acinetobacter*; MDRP, multi-drug-resistant *Pseudomonas aeruginosa*; MRSA, methicillin-resistant *Staphylococcus aureus*, VRE, vancomycin-resistant *Enterococcus*).

The time-dependent inactivation profiles of AMRB and AMSB in WWTP wastewater during UV and UV-LED treatments are presented in Figure 1. Although the inactivation time differed among the bacteria, all targeted AMRB and AMSB present in WWTP wastewater were inactivated by both UV and UV-LED. Inactivation of AMRB and AMSB by UV and UV-LED followed pseudo-first-order kinetics as previously reported for the UV disinfection of multiple bacteria and viruses [68,112,113,114]. The majority of the AMRB (CRE, ESBL-E, MDRA, MDRP, and VRE) and AMSB (*Acinetobacter*, *Enterococccus*, *E. coli*, and *P. aeruginosa*) with the exception of MRSA and *S. aureus* in the WWTP wastewater were rapidly inactivated at the level of >90% after 0.3 min, and this level reached >99% after 0.5 min UV treatment. In the case of the UV-LED treatment, the time required for inactivation tended to be longer than that for the UV treatment; however, the same inactivation effect on microorganisms was observed with the UV treatment. No significant differences (*p* < 0.05) were observed in the effects of UV irradiation on the AMRB and AMSB. The inactivation of MRSA and *S. aureus* progressed more slowly than did that of other microorganisms in both UV and UV-LED treatments and required 1 min for UV and greater than 3 min for UV-LED to achieve >90% inactivation. The rigidity of the cell wall structure of MRSA and *S. aureus* may be the likely reason for their resistance to UV inactivation, as the cell walls of MRSA and *S. aureus* are stronger than those of other bacteria. This makes them resistant to multiple environmental conditions [115,116] and chlorine disinfection [117,118].

**Figure 2:**
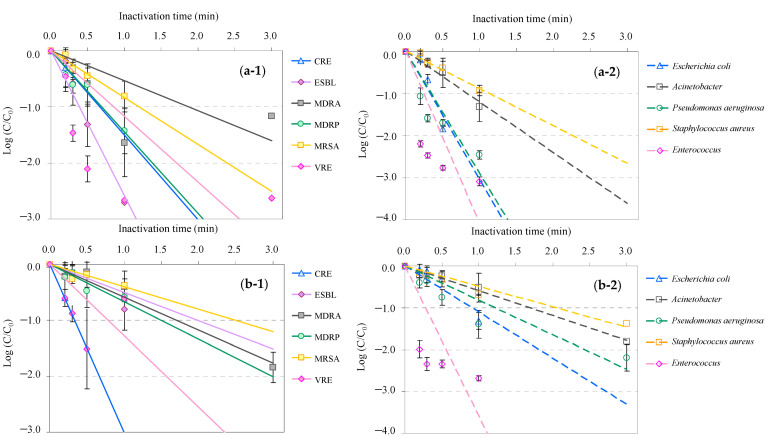
Relative antimicrobial-resistant bacteria (AMRB) and antimicrobial-susceptible bacteria (AMSB) under UV and UV-LED treatment of hospital effluent: (**a-1**) UV for AMRB, (**a-2**) UV for AMSB, (**b-1**) UV-LED for AMRB, and (**b-2**) UV-LED for AMSB (C_0_, initial bacterial counts; C, bacterial counts after treatment).

The inactivation profiles of AMRB and AMSB in hospital effluent during UV and UV-LED treatment are presented in Figure 2. When UV or UV-LED disinfection was applied to the hospital effluent, the time required for inactivation tended to increase; however, the trend of inactivation for the various suborgins was similar to the results obtained with the wastewater treatment plant effluent. Greater than 90% of AMRB and AMSB with the exception of MRSA and *S. aureus* were inactivated after 0.5 min in UV treatment and after 0.5 min in UV-LED treatment, respectively. Conversely, MRSA and *S. aureus* were gradually inactivated at 54, 66, and 84% for the former and at 49, 60, and 88% for the latter in response to UV treatment and at 44, 29, and 54% for the former and at 0, 54, and 80% for the latter in response to UV-LED treatment. These inactivation levels occurred after 0.5-, 1-, and 3-min treatments and reached the level of >99% after 3 mins of treatment, respectively. Interestingly, the inactivation effect obtained in this study tended to be mainly microorganisms that fit the regression curve, whereas in the case of Enterococcus, the fit to the straight line was less pronounced. These results would be due to the fact that *Enterococcus* is composed of a complex of many different microorganisms and has different characteristics compared to other microorganisms, which tends to deviate the inactivation system from a simple exponential linear system. This trend was also observed in a similar study on the inactivation of microorganisms in wastewater [119,120]. Therefore, it will be a challenge to try to analyze the models and mechanisms of the inactivation reactions for each microorganism in the future.

These results demonstrate the effectiveness of UV inactivation of AMRB and AMSB in both WWTP and hospital wastewater. The mechanism of inactivation of microorganisms by UV is considered to be mainly due to inhibition of gene transcription by dimerization of nucleobases in microbial DNA [121,122,123]. On the other hand, the inactivation mechanisms need to be elucidated at the molecular level due to the various structural and biological differences among microorganisms. Further developments need to be addressed for the inactivation mechanism of AMRB and AMSB with different wavelengths of ultraviolet light including UV-LEDs in the near future.

### 3.2. Inactivation Kinetics of AMRB and AMSB in the WWTP Wastewater and Hospital Effluent by UV and UV-LED Treatment

The distribution of the inactivation rates for AMRB and AMSB in response to UV and UV-LED treatments is summarized in Table 3, and the estimated distribution of the half-lives of the antimicrobials is presented in Table S2. The mean inactivation rate constants for AMRB and AMSB in WWTP wastewater were 6.6 ± 2.7 min^−1^ for UV and 7.2 ± 2.8 min^−1^ for UV-LED, and in hospital effluent, they were 3.2 ± 1.6 min^−1^ and 4.0 ± 1.8 min^−1^, respectively. Additionally, the mean inactivation rate constants for AMRB and AMSB in hospital effluent were 3.9 ± 2.7 min^−1^ for UV and 4.8 ± 2.4 min^−1^ for UV-LED, and in hospital effluent, these values were 3.4 ± 4.3 min^−1^ and 2.2 ± 0.9 min^−1^, respectively. The estimated half-lives typically ranged from <0.1 to 0.9 min. Detailed distributions of the half-lives of AMRB and AMSB are summarized in Table S2.

When comparing the inactivation effects of UV and UV-LED treatments, statistically significant differences (*p* < 0.05) were observed for MDRP, *P. aeruginosa*, *E. coli*, and *Enterococccus* in WWTP wastewater, and ESBL and *P. aeruginosa* in hospital effluent, but not for other microorganisms. Table 4 summarizes the estimated fluences required to inactivate 99% of each microorganism based on the inactivation of AMRB and AMSB over time. The results revealed that the fluences required for inactivation of AMRB and AMSB were 16 ± 6 mJ/cm^2^ and 14 ± 10 mJ/cm^2^ for UV and 37 ± 22 mJ/cm^2^ and 30 ± 17 mJ/cm^2^ for UV-LED for WWTP wastewater and 10 ± 5 mJ/cm^2^ and 8 ± 5 mJ/cm^2^ for UV and 31 ± 22 mJ/cm^2^ and 23 ± 12 mJ/cm^2^ for UV-LED for hospital effluent, respectively.

A trend of 1.5- to 2-fold higher fluences required for the inactivation of different microorganisms was observed for UV-LED compared to that for UV. However, statistically significant differences (*p* < 0.05) were observed between the two for the fluences required for the inactivation of MDRA and *P. aeruginosa* in WWTP wastewater and MDRP and *Enterococcus* in hospital effluent, thus suggesting that the characteristic differences in susceptibility attributable to bacterial species between UV and UV-LED light tended to be small. The technological development of diodes that are used to make UV-LEDs has progressed rapidly worldwide, and it is expected that UV irradiation will become possible with a much higher output power [32,45,124]. The efficacy and effectiveness of UV-LEDs remain largely unknown, and further detailed studies are required before these sources can be of practical use [30,40,125]. The results of this study are of interest in the context of the investigation of low-energy, long-life UV-LEDs as mercury-free alternatives to UV light. In addition, the fluences required for the inactivation of AMRB and AMSB will help us to evaluate the treatment condition for effective inactivation of AMRB and AMSB in wastewater. The present results support the need for further conclusive research that considers experimental, technical, and regional customs, biases, and other unknown factors.

### 3.3. Removal of Antimicrobial-Resistance Genes in WWTP Wastewater and Hospital Effluent by UV and UV-LED Treatment

**Figure 3:**
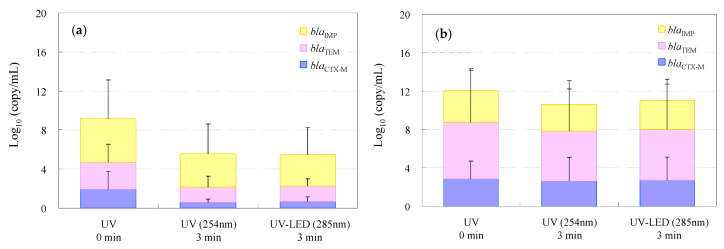
Detected numbers of CRE and ESBL-E as AMRGs in WWTP wastewater (**a**) and the hospital effluent (**b**). (AMRGs: antimicrobial-resistance genes).

The time-dependent resistome profiles of WWTP and hospital wastewater during UV and UV-LED treatment are summarized in Figure 3. All antimicrobial-resistance gene (AMRG) species were detected from the wastewater before treatment, and the mean numbers of AMRGs were 4.5 ± 4.0 log_10_ (copy/mL) for *bla*_IMP_, 2.7 ± 1.9 log_10_ (copy/mL) for *bla*_TEM_, and 1.9 ± 1.8 log_10_ (copy/mL) for *bla*_CTX-M_ in WWTP wastewater and 3.3 ± 2.3 log_10_ (copy/mL) for *bla*_IMP_, 5.9 ± 5.4 log_10_ (copy/mL) for *bla*_TEM_, and 2.9 ± 1.8 log_10_ (copy/mL) for *bla*_CTX-M_ in hospital effluent, respectively (Table S3).

UV and UV-LED processes tended to remove AMRGs with removal rates of 1.1 log_10_ for *bla*_IMP_, 1.1 log_10_ for *bla*_TEM_, and 1.4 log_10_ for *bla*_CTX-M_ in UV, while for the UV-LED processes, the removal rate was 1.3 log_10_ for *bla*_IMP_, 1.2 log_10_ for *bla*_TEM_, and 1.3 log_10_ for *bla*_CTX-M_ in WWTP wastewater. In hospital effluent, removal rates of AMRGs were 0.5 log_10_ for *bla*_IMP_, 0.7 log_10_ for *bla*_TEM_, 0.2 log_10_ for *bla*_CTX-M_ in UV, and 0.3 log_10_ for *bla*_IMP_, 0.6 log_10_ for *bla*_TEM_, and 0.1 log_10_ for *bla*_CTX-M_ in UV-LED processes, respectively. Notably, there was no statistically significant decrease (*p* < 0.05) in AMRGs between the UV and UV-LED treatments, although the DNA of AMRB that did not survive in the water samples was accounted for according to detected genes [126,127]. In contrast, none of the AMRGs targeted in this study were completely removed, and some tended to remain after the treatment. Previously reported ozone treatment was effective in treating both viable AMRB and AMRGs as genes, the reason would be related to the strong oxidizing potential of ozone, which is not seen in UV and UV-LED treatment [128,129,130]. This will be an important issue when considering the environmental impacts of AMRGs [131,132,133].

**Figure 4:**
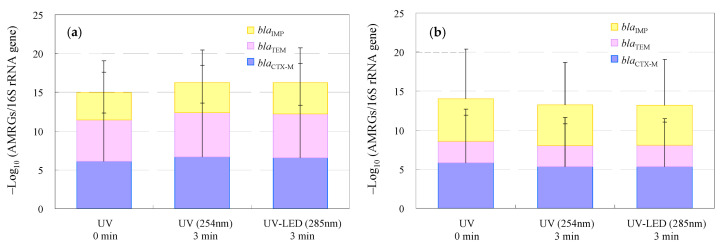
Relative resistome profiles of CRE and ESBL-E as AMRGs in the WWTP wastewater (**a**) and the hospital effluent (**b**). (AMRGs: antimicrobial-resistance genes).

The relative abundances of the resistome profiles of each wastewater sample are presented in Figure 4 and Table S3. The distribution of genes corresponding to the different AMRGs in the effluent before treatment was −5.4 ± −6.4 log_10_ (AMRGs/16S rRNA gene) for *bla*_IMP_, 2.8 ± 3.3 log_10_ (AMRGs/16S rRNA gene) for *bla*_TEM_, and 5.8 ± 6.9 log_10_ (AMRGs/16S rRNA gene) for *bla*_CTX-M_ genes. These results were similar to those obtained abroad (−5 to −1 log_10_ [AMRGs/16S rRNA gene]) [134,135,136]. AMRGs after various types of ozone treatment for *bla*_IMP_, *bla*_TEM_, and *bla*_CTX-M_ in response to UV were −3.9 ± −4.2, −5.7 ± −6.2, and −6.7 ± −7.0 and in response to UV-LED were −4.0 ± −4.5, −5.7 ± −6.5, and −6.6 ± −6.8 for WWTP wastewater, and these values were −5.2 ± −5.4, −2.7 ± −3.6, and −5.3 ± −5.5 and −5.1 ± −5.9, −2.8 ± −3.4, and −5.3 ± −5.7 for hospital effluent, respectively. These results suggest that although AMRGs are removed by UV-based treatments, they remain in the river environment with less significant changes in their morphology [132,137]. The DNA of AMRB that did not survive in the water samples was accounted for by viable AMRB as detected genes [126,127]. The present results support the need for further conclusive research that considers experimental, technical, and regional customs, biases, and other unknown factors.

Several researchers have been concerned regarding the persistence of AMRGs in conventional wastewater treatment processes such as biological treatment and chlorine disinfection in wastewater treatment plants (WWTPs) and water environments. [68,137,138,139,140]. The potential effects of AMRGs in an aqueous environment are due to a wide variety of microorganisms in activated sludge in biological treatment reactors at WWTPs [141], and there have been concerns that AMRB could result in a pool of antimicrobial-resistant bacteria through zygotic transmission or transformation [94,142,143]. Additionally, the effect of gene transfer and transformation on the potential emergence of new AMRB must be considered [144,145,146]. Therefore, detailed and quantitative assessments of environmental risks to ecosystems and human health risks from both AMRB and AMRGs in river environments are considered important issues in the near future [147,148,149,150,151].

One way to overcome these challenges is to introduce the types of advanced water treatment systems that have been examined in this study. Additionally, there have been a growing number of studies in recent years focused on reducing or eliminating the environmental impact of antimicrobials, AMRB, and AMSB in wastewater discharged into public sewers by on-site treatment at hospital facilities before they are aggregated and diluted for discharge into sewers [1,20,117]. Recently, in addition to the UV treatments covered in this study, UV chlorine [123,152], ozone [73,153], electrochemical [154,155], peracetic acid [156,157], and membrane [158,159] treatments appear to be effective in regard to decreasing the levels of these new environmental pollutants in wastewater discharged into the water environment.

### 3.4. Bacterial Community Structure Analysis

**Figure 5:**
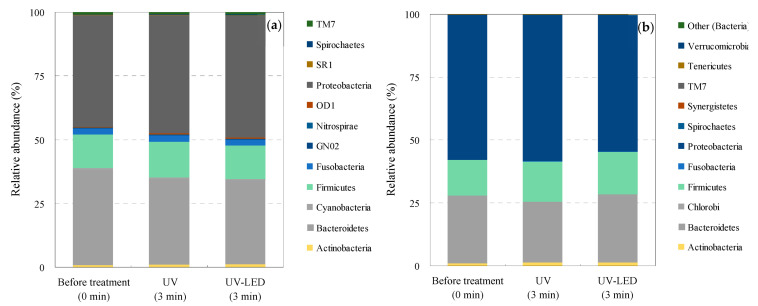
Taxonomic diversity of bacterial communities in the direct UV and UV-LED treated wastewater samples. ((**a**) WWTP wastewater and (**b**) hospital effluent).

The bacterial community structures in the WWTP wastewater and hospital effluent before and after UV and UV-LED treatment based on the taxonomic affiliation of the OTUs are summarized in Figure 5. A total of 147,940, 154,719, and 156,556 bacterial 16S rRNA reads were obtained from WWTP wastewater upon commencement of UV and UV-LED treatment, respectively (7222 OTUs in total). From the hospital effluent, 140,437, 125,587, and 121,287 reads were collected from the water samples upon the commencement of treatment and after UV and UV-LED treatments, respectively (2954 OTUs in total).

WWTP wastewater contained 42 bacterial phyla, 132 classes, 232 orders, 395 families, and 768 genera. The hospital effluent contained 25 bacterial phyla, 50 classes, 91 orders, 172 families, and 385 genera. Additionally, the major phyla were *Proteobacteria* (44%), *Bacteroidetes* (38%), *Firmicutes* (13%), *Fusobacteria* (2%), and *TM7* (0.8%) in WWTP wastewater and *Proteobacteria* (58%), *Bacteroidetes* (27%), *Firmicutes* (14%), *Actinobacteria* (1%), and TM7 (0.1%) in hospital effluent. *Proteobacteria* comprise the majority of the composition of both WWTP wastewater and hospital effluents and are considered to be one of the most diverse and abundant groups of microbes on earth with low pathogenic potential [160]. This finding suggests that environmental bacteria comprise the majority of microorganisms when considering hospital wastewater as a whole and that microorganisms such as AMRB that are pathogenic and infectious and require clinical attention comprise only a small proportion [12,23]. Conversely, *Bacteroides* is a biotactic anaerobic gram-negative bacillus that is endemic to the human colon. It is also an opportunistic infectious organism that causes intra-abdominal abscesses and septicemia in susceptible hosts, and it can survive in aquatic environments [161,162].

Bacterial community structure was not apparently affected during both UV and UV-LED treatment for the WWTP wastewater (UV: *Proteobacteria* [46%], *Bacteroidetes* [34%], *Firmicutes* [14%], *Fusobacteria* [2%], and *Actinobacteria* [1%]; UV-LED: *Proteobacteria* [48%], *Bacteroidetes* [33%], *Firmicutes* [13%], *Fusobacteria* [2%], and *Actinobacteria* [1%]) and the hospital effluent (UV: *Proteobacteria* [58%], *Bacteroidetes* [24%], *Firmicutes* [16%], *Actinobacteria* [1%], and TM7 [0.1%]; UV-LED: *Proteobacteria* [54%], *Bacteroidetes* [27%], *Firmicutes* [17%], *Actinobacteria* [1%], and TM7 [0.1%]). Overall, the present results suggest the importance of introducing advanced wastewater treatment for the removal of AMRB and AMRGs, although some bacteria are not completely removed [163]. This appears to be reasonable considering the existence of multiple microorganisms [138,163,164] and AMRGs [165,166,167,168] in wastewater and river water. The results of the present study suggest that comprehensive removal of AMRB, including AMRGs, requires the introduction of advanced wastewater treatment systems when considering multiple microorganisms [138,163,164] and AMRGs [165,166,167,168].

Recent research has provided insights into the environmental risk of both AMRB and AMRGs [169,170,171]. The environmental risk of infection by AMRB in water and via the ecosystem and the further development of AMRB in the presence of residual antimicrobials or AMRGs in water are currently being assessed [148,149]. Furthermore, the present study provides valuable information for preventing infectious diseases in aquatic environments, including wastewater. To the best of our knowledge, this is the first report demonstrating the inactivation profiles of AMRB and AMRGs in hospital wastewater using direct UV and UV-LED treatments. These results will improve the understanding of the environmental pollution associated with AMRB and AMRGs in aquatic environments. The introduction of any advanced wastewater treatment system that can be used in conjunction with UV radiation such as ozonation at medical facilities and WWTPs may provide a one-step forward measure for the reduction of any risks caused by pollution in aquatic environments.

## 4. Conclusions

This study investigated the effectiveness of UV and UV-LED treatments for the inactivation of AMRB and AMRGs in model WWTP wastewater and real hospital wastewater. Overall, the results indicate that direct UV and UV-LED treatments inactivated the majority of AMRB and partially removed AMRGs. No significant differences were observed in the effects of UV irradiation on genes that comprise these microorganisms, and the taxonomic diversity of microorganisms did not change even after additional disinfection with UV. Additional advanced wastewater treatments are necessary to completely remove AMRGs. The finding that AMRB and AMRGs that could impact the environment were effectively inactivated and/or removed during treatment could be a countermeasure to mitigate the environmental and human health impacts associated with the prevalence of AMR. The addition of the present on-site UV disinfection treatment system may be effective not only in regional places but also in developing regions and countries, due to the simplicity and convenience of preventing the potential spread of infectious diseases at the upstream stage in terms of drainage hygiene. Economic advantages are important considerations when developing practical applications. Further research is urgently required to prevent the spread of infectious diseases from wastewater. The overall results provide a better understanding of the current situation of AMR in hospital wastewater and provide insights for devising strategies to eliminate or mitigate the burden of AMRB flow into aquatic environments.

## Figures and Tables

**Table 1:** List of target antimicrobial-resistant bacteria (AMRB) and antimicrobial-susceptible bacteria (AMSB).

Classification	Bacteria	Abbreviation
AMRB	Carbapenem-resistant *Enterobacterales*	CRE
	Extended-spectrum *β*-lactamase (ESBL)-producing *Enterobacterales*	ESBL-E
	Multi-drug-resistant *Acinetobacter*	MDRA
	Multi-drug-resistant *Pseudomonas aeruginosa*	MDRP
	Methicillin-resistant *Staphylococcus aureus*	MRSA
	Vancomycin-resistant *Enterococcus*	VRE
AMSB	*Acinetobacter*	*Acinetobacter*
	*Enterococcus*	*Enterococcus*
	*Escherichia col*	*E. col* *i*
	*Pseudomonas aeruginosa*	*P. aeruginosa*
	*Staphylococcus aureus*	*S. aureus*

**Table 2:** The occurrence of AMRB and AMSB in the wastewater treatment plant (WWTP) influent, secondary effluent, effluent, model WWTP wastewater, and hospital effluent.

Bacteria	Bacteria Counts (CFU/mL)				
	WWTP Influent	WWTP Secondary Effluent *	WWTP Effluent **	WWTP Wastewater ***	Hospital Effluent
CRE	131	6	16	42	78
ESBL-E	963	10	9	73	131
MDRA	236	17	17	172	81
MDRP	43	N.D.	1	9	215
MRSA	47	7	6	21	31
VRE	35	N.D.	N.D.	27	151
*Acinetobacter*	257	34	15	91	307
*Enterococcus*	4267	664	140	1163	5736
*Escherichia coli*	20,000	145	5	3000	13,000
*Pseudomonas aeruginosa*	50	N.D.	1	21	1096
*Staphylococcus aureus*	126	8	17	24	63

(CRE, carbapenem-resistant *Enterobacterales*; ESBL-E, extended-spectrum *β*-lactamase-producing *Enterobacterales*; MDRA, multidrug-resistant *Acinetobacter*; MDRP, multidrug-resistant *Pseudomonas aeruginosa*; MRSA, methicillin-resistant *Staphylococcus aureus*; VRE, vancomycin-resistant *Enterococcus*; N.D., not detected). * Water was obtained from a conventional activated sludge (CAS) system in a WWTP. ** Water after a combined treatment with CAS and chlorine. *** Prepared by mixing WWTP influent and WWTP secondary effluent (1:9 [*v*/*v*]).

**Table 3:** Reaction rate constants for AMRB and AMSB during UV (254 nm) and UV-LED (280 nm) treatment of WWTP wastewater and hospital effluent.

Bacteria	Inactivation Rate (min^−1^)			
	WWTP Wastewater		Hospital Effluent	
	UV (254 nm)	UV-LED (280 nm)	UV (254 nm)	UV-LED (280 nm)
CRE	5.794	2.351	6.932	11.992
ESBL-E	6.916	3.606	6.711	1.292
MDRA	8.440	4.763	1.497	1.511
MDRP	4.558	1.407	3.346	1.479
MRSA	3.215	1.663	1.928	0.804
VRE	10.649	5.417	2.985	3.554
*Acinetobacter*	8.920	5.126	2.524	1.465
*Enterococcus*	6.819	2.582	5.306	3.507
*Escherichia coli*	8.360	4.613	7.254	2.517
*Pseudomonas aeruginosa*	9.615	6.027	6.674	1.928
*Staphylococcus aureus*	2.501	1.601	2.047	1.506

**Table 4:** Fluence required for 99% inactivation of AMRB and AMSB during UV (254 nm) and UV-LED (280 nm) treatment of WWTP wastewater and hospital effluent.

Bacteria	Fluences Required for Inactivation (mJ/cm^2^) (Mean (SD))			
	WWTP Wastewater		Hospital Effluent	
	UV (254 nm)	UV-LED (280 nm)	UV (254 nm)	UV-LED (280 nm)
CRE	17.2 (3.2)	34.3 (30.3)	5.1 (0.8)	9.6 (4.5)
ESBL-E	15.0 (3.5)	26.5 (11.4)	5.4 (0.5)	31.9 (11.1)
MDRA	11.6 (2.4)	18.7 (0.9)	14.9 (11.7)	32.8 (4)
MDRP	14.6 (3.3)	63.2 (35.3)	10.3 (3.9)	29.3 (3.9)
MRSA	26.9 (1.7)	65.2 (34.6)	17.8 (6.8)	70.6 (58)
VRE	8.7 (9.0)	16.4 (4.9)	7.2 (4.1)	10.4 (3.3)
*Acinetobacter*	9.0 (0.3)	17.2 (1.2)	11.4 (1.3)	40.8 (15.5)
*Enterococcus*	11.8 (0.5)	35 (2.9)	4.2 (1.7)	12.7 (0.5)
*Escherichia coli*	9.5 (0.2)	25.9 (8)	6.0 (2.3)	16.7 (5.9)
*Pseudomonas aeruginosa*	8.2 (1.1)	14.4 (1.5)	4.7 (0.1)	14.1 (3.2)
*Staphylococcus aureus*	32.7 (0.0)	55.7 (4.6)	15.7 (3.0)	28.3 (3.2)

## Data Availability

The data are not publicly available due to privacy or ethical restrictions.

## Supplementary Materials

The following supporting information can be downloaded from https://www.mdpi.com/article/10.3390/antibiotics13080711/s1, Figure S1. UV inactivation reactor used for the evaluation. ([a]: UV system, [b]: UV-LED system); Table S1. Primers and PCR conditions for AMRGs analyses; Table S2. Half-life of each antimicrobial during UV and UV-LED treatment of WWTP wastewater and hospital effluent; Table S3. Occurrence of AMRGs in WWTP wastewater and hospital effluent.

## References

[1] Khan N.A., Vambol V., Vambol S., Bolibrukh B., Sillanpaa M., Changani F., Esrafili A., Yousefi M. (2021). Hospital effluent guidelines and legislation scenario around the globe: A critical review. J. Environ. Chem. Eng..

[2] Cameron A., Esiovwa R., Connolly J., Hursthouse A., Henriquez F. (2022). Antimicrobial resistance as a global health threat: The need to learn lessons from the COVID-19 pandemic. Glob. Policy.

[3] Wang Y., Han Y., Li L., Liu J., Yan X. (2022). Distribution, sources, and potential risks of antibiotic resistance genes in wastewater treatment plant: A review. Environ. Pollut..

[4] World Health Organization (WHO) (2014). Antimicrobial Resistance: Global Report on Surveillance 2014.

[5] Centers for Disease Control and Prevention (CDC) (2019). Antibiotic Resistance Threats in the United States.

[6] Jim O.N. (2014). Antimicrobial resistance: Tackling a crisis for the health and wealth of nations. Rev. Antimicrob. Resist..

[7] Murray C.J.L., Ikuta K.S., Sharara F., Swetschinski L., Robles Aguilar G., Gray A., Han C., Bisignano C., Rao P., Wool E. (2022). Global burden of bacterial antimicrobial resistance in 2019: A systematic analysis. Lancet.

[8] The Government of Japan (2016). National Action Plan on Antimicrobial Resistance (AMR), 2016.

[9] The Government of Japan (2023). National Action Plan on Antimicrobial Resistance (AMR), 2023.

[10] Al Salah D.M.M., Ngweme G.N., Laffite A., Otamonga J.P., Mulaji C., Poté J. (2020). Hospital wastewaters: A reservoir and source of clinically relevant bacteria and antibiotic resistant genes dissemination in urban river under tropical conditions. Ecotoxicol. Environ. Saf..

[11] Hassoun-Kheir N., Stabholz Y., Kreft J.U., de la Cruz R., Romalde J.L., Nesme J., Sørensen S.J., Smets B.F., Graham D., Paul M. (2020). Comparison of antibiotic-resistant bacteria and antibiotic resistance genes abundance in hospital and community wastewater: A systematic review. Sci. Total Environ..

[12] Sekizuka T., Tanaka R., Hashino M., Yatsu K., Kuroda M. (2022). Comprehensive genome and plasmidome analysis of antimicrobial resistant bacteria in wastewater treatment plant effluent of Tokyo. Antibiotics.

[13] Muraki Y., Kitamura M., Maeda Y., Kitahara T., Mori T., Ikeue H., Tsugita M., Tadano K., Takada K., Akamatsu T. (2013). Nationwide surveillance of antimicrobial consumption and resistance to *Pseudomonas aeruginosa* isolates at 203 Japanese hospitals in 2010. Infection.

[14] Van Boeckel T.P., Gandra S., Ashok A., Caudron Q., Grenfell B.T., Levin S.A., Laxminarayan R. (2014). Global antibiotic consumption 2000 to 2010: An analysis of national pharmaceutical sales data. Lancet Infect. Dis..

[15] Stefani S., Chung D.R., Lindsay J.A., Friedrich A.W., Kearns A.M., Westh H., MacKenzie F.M. (2012). Meticillin-resistant *Staphylococcus aureus* (MRSA): Global epidemiology and harmonisation of typing methods. Int. J. Antimicrob. Agents.

[16] Lakhundi S., Zhang K. (2018). *Methicillin-resistant Staphylococcus aureus*: Molecular characterization, evolution, and epidemiology. Clin. Microbiol. Rev..

[17] Lee A.S., de Lencastre H., Garau J., Kluytmans J., Malhotra-Kumar S., Peschel A., Harbarth S. (2018). Methicillin-resistant *Staphylococcus aureus*. Nature Rev. Dis. Primers.

[18] Shiozaki Y. (2018). The action for the antimicobial resistance issue in Japan. Jpn. Assoc. Infect. Dis..

[19] Khan M.T., Shah I.A., Ihsanullah I., Naushad M., Ali S., Shah S.H.A., Mohammad A.W. (2021). Hospital wastewater as a source of environmental contamination: An overview of management practices, environmental risks, and treatment processes. J. Water Proc. Eng..

[20] Verlicchi P. (2021). Trends, new insights and perspectives in the treatment of hospital effluents. Curr. Opin. Environ. Sci. Health.

[21] Pariente M.I., Segura Y., Álvarez-Torrellas S., Casas J.A., de Pedro Z.M., Diaz E., García J., López-Muñoz M.J., Marugán J., Mohedano A.F. (2022). Critical review of technologies for the on-site treatment of hospital wastewater: From conventional to combined advanced processes. J. Environ. Manag..

[22] Pruden A., Vikesland P.J., Davis B.C., de Roda Husman A.M. (2021). Seizing the moment: Now is the time for integrated global surveillance of antimicrobial resistance in wastewater environments. Curr. Opin. Microbiol..

[23] Sekizuka T., Itokawa K., Tanaka R., Hashino M., Yatsu K., Kuroda M. (2022). Metagenomic analysis of urban wastewater treatment plant effluents in tokyo. Infect. Drug Resist..

[24] Rekhate C.V., Srivastava J.K. (2020). Recent advances in ozone-based advanced oxidation processes for treatment of wastewater- A review. Chem. Eng. J. Adv..

[25] Bhandari G., Chaudhary P., Gangola S., Gupta S., Gupta A., Rafatullah M., Chen S. (2023). A review on hospital wastewater treatment technologies: Current management practices and future prospects. J. Water Proc. Eng..

[26] Adeoye J.B., Tan Y.H., Lau S.Y., Tan Y.Y., Chiong T., Mubarak N.M., Khalid M. (2024). Advanced oxidation and biological integrated processes for pharmaceutical wastewater treatment: A review. J. Environ. Manag..

[27] Amin N., Foster T., Shimki N.T., Willetts J. (2024). Hospital wastewater (HWW) treatment in low- and middle-income countries: A systematic review of microbial treatment efficacy. Sci. Total Environ..

[28] Saravanan A., Deivayanai V.C., Kumar P.S., Rangasamy G., Hemavathy R.V., Harshana T., Gayathri N., Alagumalai K. (2022). A detailed review on advanced oxidation process in treatment of wastewater: Mechanism, challenges and future outlook. Chemosphere.

[29] Iqbal J., Shah N.S., Ali Khan J., Naushad M., Boczkaj G., Jamil F., Khan S., Li L., Murtaza B., Han C. (2024). Pharmaceuticals wastewater treatment via different advanced oxidation processes: Reaction mechanism, operational factors, toxicities, and cost evaluation—A review. Sep. Purif. Technol..

[30] Li X., Cai M., Wang L., Niu F., Yang D., Zhang G. (2019). Evaluation survey of microbial disinfection methods in UV-LED water treatment systems. Sci. Total Environ..

[31] Ryan U., Hill K., Deere D. (2022). Review of generic screening level assumptions for quantitative microbial risk assessment (qmra) for estimating public health risks from australian drinking water sources contaminated with *Cryptosporidium* by recreational activities. Water Res..

[32] Matafonova G., Batoev V. (2018). Recent advances in application of UV light-emitting diodes for degrading organic pollutants in water through advanced oxidation processes: A review. Water Res..

[33] Nguyen T.M.H., Suwan P., Koottatep T., Beck S.E. (2019). Application of a novel, continuous-feeding ultraviolet light emitting diode (UV-LED) system to disinfect domestic wastewater for discharge or agricultural reuse. Water Res..

[34] Song K., Mohseni M., Taghipour F. (2016). Application of ultraviolet light-emitting diodes (UV-LEDs) for water disinfection: A review. Water Res..

[35] Pelayo D., Rivero M.J., Santos G., Gómez P., Ortiz I. (2023). Techno-economic evaluation of UV light technologies in water remediation. Sci. Total Environ..

[36] Li G.Q., Wang W.L., Huo Z.Y., Lu Y., Hu H.Y. (2017). Comparison of UV-LED and low pressure UV for water disinfection: Photoreactivation and dark repair of *Escherichia coli*. Water Res..

[37] Nyangaresi P.O., Qin Y., Chen G., Zhang B., Lu Y., Shen L. (2019). Comparison of UV-LED photolytic and UV-LED/TiO_2_ photocatalytic disinfection for *escherichia coli* in water. Catal. Today.

[38] Wan Q., Cao R., Wen G., Xu X., Xia Y., Wu G., Li Y., Wang J., Xu H., Lin Y. (2022). Efficacy of UV-LED based advanced disinfection processes in the inactivation of waterborne fungal spores: Kinetics, photoreactivation, mechanism and energy requirements. Sci. Total Environ..

[39] Dulin D., Mill T. (1982). Development and evaluation of sunlight actinometers. Environ. Sci. Technol..

[40] Chen J., Loeb S., Kim J.H. (2017). Led revolution: Fundamentals and prospects for UV disinfection applications. Environ. Sci. Water Res. Technol..

[41] Ma B., Seyedi S., Wells E., McCarthy D., Crosbie N., Linden K.G. (2022). Inactivation of biofilm-bound bacterial cells using irradiation across UVC wavelengths. Water Res..

[42] Bae J.Y., Kim Y., Kim H., Kim Y., Jin J., Bae B.S. (2015). Ultraviolet light stable and transparent sol–gel methyl siloxane hybrid material for UV light-emitting diode (UV LED) encapsulant. ACS Appl. Mater. Interfaces.

[43] Gao Z.C., Lin Y.L., Xu B., Xia Y., Hu C.Y., Cao T.C., Zou X.Y., Gao N.Y. (2019). Evaluating iopamidol degradation performance and potential dual-wavelength synergy by UV-LED irradiation and UV-LED/chlorine treatment. Chem. Eng. J..

[44] Cai A., Deng J., Ye C., Zhu T., Ling X., Shen S., Guo H., Li X. (2022). Highly efficient removal of deet by UV-LED irradiation in the presence of iron-containing coagulant. Chemosphere.

[45] Song K., Taghipour F., Mohseni M. (2019). Microorganisms inactivation by wavelength combinations of ultraviolet light-emitting diodes (UV-LEDs). Sci. Total Environ..

[46] Zou X.Y., Lin Y.L., Xu B., Cao T.C., Tang Y.L., Pan Y., Gao Z.C., Gao N.-Y. (2019). Enhanced inactivation of *E. coli* by pulsed UV-LED irradiation during water disinfection. Sci. Total Environ..

[47] Pousty D., Hofmann R., Gerchman Y., Mamane H. (2021). Wavelength-dependent time–dose reciprocity and stress mechanism for UV-LED disinfection of *Escherichia coli*. J. Photochem. Photobiol. B.

[48] Gerchman Y., Mamane H., Friedman N., Mandelboim M. (2020). UV-LED disinfection of coronavirus: Wavelength effect. J. Photochem. Photobiol. B.

[49] Inagaki H., Saito A., Kaneko C., Sugiyama H., Okabayashi T., Fujimoto S. (2021). Rapid inactivation of SARS-CoV-2 variants by continuous and intermittent irradiation with a deep-ultraviolet light-emitting diode (DUV-LED) device. Pathogens.

[50] Verlicchi P. (2017). Hospital Wastewaters: Characteristics, Management, Treatment and Environmental Risks.

[51] Khan N.A., Ahmed S., Farooqi I.H., Ali I., Vambol V., Changani F., Yousefi M., Vambol S., Khan S.U., Khan A.H. (2020). Occurrence, sources and conventional treatment techniques for various antibiotics present in hospital wastewaters: A critical review. Trends Anal. Chem..

[52] Fatimazahra S., Latifa M., Laila S., Monsif K. (2023). Review of hospital effluents: Special emphasis on characterization, impact, and treatment of pollutants and antibiotic resistance. Environ. Monit. Assess..

[53] Haeusser S., Weber M., Mauer C., Linnemann V., Pfannstiel A., Pinnekamp J., Wintgens T., Klümper C., Beier S. (2023). On-site treatment of hospital wastewater in a full-scale treatment plant in Germany: SARS-CoV-2 and treatment performance. Water Sci. Technol..

[54] Gutierrez M., Mutavdžić Pavlović D., Stipaničev D., Repec S., Avolio F., Zanella M., Verlicchi P. (2024). A thorough analysis of the occurrence, removal and environmental risks of organic micropollutants in a full-scale hybrid membrane bioreactor fed by hospital wastewater. Sci. Total Environ..

[55] Yu S.Y., Xie Z.H., Wu X., Zheng Y.Z., Shi Y., Xiong Z.K., Zhou P., Liu Y., He C.-S., Pan Z.C. (2024). Review of advanced oxidation processes for treating hospital sewage to achieve decontamination and disinfection. Chin. Chem. Lett..

[56] Azuma T., Uchiyama T., Zhang D., Usui M., Hayashi T. (2022). Distribution and characteristics of carbapenem-resistant and extended-spectrum *β*-lactamase (ESBL) producing *Escherichia coli* in hospital effluents, sewage treatment plants, and river water in an urban area of Japan. Sci. Total Environ..

[57] Japan Meteorological Agency Weather Statistics. http://www.jma.go.jp/jma/index.html.

[58] Zheng J., Su C., Zhou J., Xu L., Qian Y., Chen H. (2017). Effects and mechanisms of ultraviolet, chlorination, and ozone disinfection on antibiotic resistance genes in secondary effluents of municipal wastewater treatment plants. Chem. Eng. J..

[59] Dunkin N., Weng S., Coulter C.G., Jacangelo J.G., Schwab K.J. (2018). Impacts of virus processing on human norovirus GI and GII persistence during disinfection of municipal secondary wastewater effluent. Water Res..

[60] Nyangaresi P.O., Qin Y., Chen G., Zhang B., Lu Y., Shen L. (2018). Effects of single and combined UV-LEDs on inactivation and subsequent reactivation of *E.coli* in water disinfection. Water Res..

[61] Keshavarzfathy M., Malayeri A.H., Mohseni M., Taghipour F. (2020). UV-LED fluence determination by numerical method for microbial inactivation studies. J. Photochem. Photobiol. A.

[62] U.S. Environmental Protection Agency (EPA) (1998). Fate, Transport and Transformation: Test Guidelines OPPTS 835.2210.

[63] U.S. Environmental Protection Agency (EPA) (1998). Fate, Transport and Transformation: Test Guidelines OPPTS 835.2240.

[64] OECD (2008). Phototransformation of Chemicals in Water-Direct Photolysis, OECD Guideline for the Testing of Chemicals No. 316.

[65] Japan Sewage Works Association (2022). Statistics of Sewerage.

[66] Azuma T., Hayashi T. (2021). Effects of natural sunlight on antimicrobial-resistant bacteria (AMRB) and antimicrobial-susceptible bacteria (AMSB) in wastewater and river water. Sci. Total Environ..

[67] Hijnen W.A.M., Beerendonk E.F., Medema G.J. (2006). Inactivation credit of UV radiation for viruses, bacteria and protozoan (oo)cysts in water: A review. Water Res..

[68] He H., Zhou P., Shimabuku K.K., Fang X., Li S., Lee Y., Dodd M.C. (2019). Degradation and deactivation of bacterial antibiotic resistance genes during exposure to free chlorine, monochloramine, chlorine dioxide, ozone, ultraviolet light, and hydroxyl radical. Environ. Sci. Technol..

[69] World Health Organization (WHO) (2015). Global Action Plan on Antimicrobial Resistance.

[70] World Health Organization (WHO) Antibiotic-Resistant “Priority Pathogens”—A Catalogue of 12 Families of Bacteria that Pose the Greatest Threat to Human Health. http://www.who.int/mediacentre/news/releases/2017/bacteria-antibiotics-needed/en/.

[71] Alekshun M.N., Levy S.B. (2007). Molecular mechanisms of antibacterial multidrug resistance. Cell.

[72] Mulani M.S., Kamble E.E., Kumkar S.N., Tawre M.S., Pardesi K.R. (2019). Emerging strategies to combat eskape pathogens in the era of antimicrobial resistance: A review. Front. Microbiol..

[73] Azuma T., Usui M., Hayashi T. (2022). Inactivation of antibiotic-resistant bacteria in wastewater by ozone-based advanced water treatment processes. Antibiotics.

[74] bioMérieux (France) Manufacturer’s Protocol for chromIDTM Chromogenic Media. http://www.biomerieux.fr/diagnostic-clinique/milieux-de-culture.

[75] Lamba M., Graham D.W., Ahammad S.Z. (2017). Hospital wastewater releases of carbapenem-resistance pathogens and genes in urban India. Environ. Sci. Technol..

[76] Glady-Croue J., Niu X.Z., Ramsay J.P., Watkin E., Murphy R.J.T., Croue J.P. (2018). Survival of antibiotic resistant bacteria following artificial solar radiation of secondary wastewater effluent. Sci. Total Environ..

[77] Haller L., Chen H., Ng C., Le T.H., Koh T.H., Barkham T., Sobsey M., Gin K.Y.H. (2018). Occurrence and characteristics of extended-spectrum *β*-lactamase- and carbapenemase- producing bacteria from hospital effluents in singapore. Sci. Total Environ..

[78] Azuma T., Otomo K., Kunitou M., Shimizu M., Hosomaru K., Mikata S., Ishida M., Hisamatsu K., Yunoki A., Mino Y. (2019). Environmental fate of pharmaceutical compounds and antimicrobial-resistant bacteria in hospital effluents, and contributions to pollutant loads in the surface waters in Japan. Sci. Total Environ..

[79] Serna-Galvis E.A., Vélez-Peña E., Osorio-Vargas P., Jiménez J.N., Salazar-Ospina L., Guaca-González Y.M., Torres-Palma R.A. (2019). Inactivation of carbapenem-resistant *Klebsiella pneumoniae* by photo-Fenton: Residual effect, gene evolution and modifications with citric acid and persulfate. Water Res..

[80] Sauter D., Stange C., Schumacher V., Tiehm A., Gnirss R., Wintgens T. (2021). Impact of ozonation and biological post-treatment of municipal wastewater on microbiological quality parameters. Environ. Sci. Water Res. Technol..

[81] Sib E., Voigt A.M., Wilbring G., Schreiber C., Faerber H.A., Skutlarek D., Parcina M., Mahn R., Wolf D., Brossart P. (2019). Antibiotic resistant bacteria and resistance genes in biofilms in clinical wastewater networks. Int. J. Hyg. Environ. Health.

[82] Schreiber C., Zacharias N., Essert S.M., Wasser F., Müller H., Sib E., Precht T., Parcina M., Bierbaum G., Schmithausen R.M. (2021). Clinically relevant antibiotic-resistant bacteria in aquatic environments—An optimized culture-based approach. Sci. Total Environ..

[83] Tsunoda R., Usui M., Tagaki C., Fukuda A., Boonla C., Anomasiri W., Sukpanyatham N., Akapelwa M.L., Nakajima C., Tamura Y. (2021). Genetic characterization of coliform bacterial isolates from environmental water in Thailand. J. Infect. Chemother..

[84] The eDNA Society (2021). Environmental DNA Sampling and Experiment Manual Ver. 2.1.

[85] Katada S., Fukuda A., Nakajima C., Suzuki Y., Azuma T., Takei A., Takada H., Okamoto E., Kato T., Tamura Y. (2021). Aerobic composting and anaerobic digestion decrease the copy numbers of antibiotic-resistant genes and the levels of lactose-degrading *Enterobacteriaceae* in dairy farms in Hokkaido, Japan. Front. Microbiol..

[86] Yang Y., Xing S., Chen Y., Wu R., Wu Y., Wang Y., Mi J., Liao X. (2021). Profiles of bacteria/phage-comediated args in pig farm wastewater treatment plants in China: Association with mobile genetic elements, bacterial communities and environmental factors. J. Hazard. Mater..

[87] Saladin M., Cao V.T.B., Lambert T., Donay J.L., Herrmann J.L., Ould-Hocine Z., Verdet C., Delisle F., Philippon A., Arlet G. (2002). Diversity of CTX-M *β*-lactamases and their promoter regions from *Enterobacteriaceae* isolated in three parisian hospitals. FEMS Microbiol. Lett..

[88] Kojima A., Ishii Y., Ishihara K., Esaki H., Asai T., Oda C., Tamura Y., Takahashi T., Yamaguchi K. (2005). Extended-spectrum-*β*-lactamase-producing *Escherichia coli* strains isolated from farm animals from 1999 to 2002: Report from the Japanese veterinary antimicrobial resistance monitoring program. Antimicrob. Age. Chemother..

[89] Wei T., Miyanaga K., Tanji Y. (2014). Persistence of antibiotic-resistant and -sensitive *Proteus mirabilis* strains in the digestive tract of the housefly (*Musca domestica*) and green bottle flies (*Calliphoridae*). Appl. Microbiol. Biotechnol..

[90] Laffite A., Al Salah D.M.M., Slaveykova V.I., Otamonga J.P., Poté J. (2020). Impact of anthropogenic activities on the occurrence and distribution of toxic metals, extending-spectra *β*-lactamases and carbapenem resistance in sub-Saharan African urban rivers. Sci. Total Environ..

[91] Yang K., Chen Q.L., Chen M.L., Li H.Z., Liao H., Pu Q., Zhu Y.G., Cui L. (2020). Temporal dynamics of antibiotic resistome in the plastisphere during microbial colonization. Environ. Sci. Technol..

[92] Ávila C., García-Galán M.J., Borrego C.M., Rodríguez-Mozaz S., García J., Barceló D. (2021). New insights on the combined removal of antibiotics and args in urban wastewater through the use of two configurations of vertical subsurface flow constructed wetlands. Sci. Total Environ..

[93] Li S., Yao Q., Liu J., Yu Z., Li Y., Jin J., Liu X., Wang G. (2022). Liming mitigates the spread of antibiotic resistance genes in an acid black soil. Sci. Total Environ..

[94] Zhang Y., Pei M., Zhang B., He Y., Zhong Y. (2021). Changes of antibiotic resistance genes and bacterial communities in the advanced biological wastewater treatment system under low selective pressure of tetracycline. Water Res..

[95] Ohore O.E., Wei Y., Wang Y., Nwankwegu A.S., Wang Z. (2022). Tracking the influence of antibiotics, antibiotic resistomes, and salinity gradient in modulating microbial community assemblage of surface water and the ecological consequences. Chemosphere.

[96] Yang Y., Li H., Wei Y., Chen Z., Chen T., Liang Y., Yin J., Yang D., Yang Z., Shi D. (2022). Comprehensive insights into profiles and bacterial sources of intracellular and extracellular antibiotic resistance genes in groundwater. Environ. Pollut..

[97] Flach C.F., Hutinel M., Razavi M., Åhrén C., Larsson D.G.J. (2021). Monitoring of hospital sewage shows both promise and limitations as an early-warning system for carbapenemase-producing *Enterobacterales* in a low-prevalence setting. Water Res..

[98] Xu S., Liu Y., Wang R., Zhang T., Lu W. (2022). Behaviors of antibiotic resistance genes (ARGs) and metal resistance genes (mrgs) during the pilot-scale biophysical drying treatment of sewage sludge: Reduction of args and enrichment of mrgs. Sci. Total Environ..

[99] Watts G.S., Youens-Clark K., Slepian M.J., Wolk D.M., Oshiro M.M., Metzger G.S., Dhingra D., Cranmer L.D., Hurwitz B.L. (2017). 16s RNA gene sequencing on a benchtop sequencer: Accuracy for identification of clinically important bacteria. J. Appl. Microbiol..

[100] Johnson J.S., Spakowicz D.J., Hong B.-Y., Petersen L.M., Demkowicz P., Chen L., Leopold S.R., Hanson B.M., Agresta H.O., Gerstein M. (2019). Evaluation of 16s rrna gene sequencing for species and strain-level microbiome analysis. Nature Commun..

[101] Deng M., Chen J., Gou J., Hou J., Li D., He X. (2018). The effect of different carbon sources on water quality, microbial community and structure of biofloc systems. Aquaculture.

[102] Quartaroli L., Silva C.M., Silva L.C.F., Lima H.S., de Paula S.O., Dias R.S., Carvalho K.B., Souza R.S., Bassin J.P., da Silva C.C. (2019). Effect of the gradual increase of salt on stability and microbial diversity of granular sludge and ammonia removal. J. Environ. Manag..

[103] Sun Z., Li G., Wang C., Jing Y., Zhu Y., Zhang S., Liu Y. (2014). Community dynamics of prokaryotic and eukaryotic microbes in an estuary reservoir. Sci. Rep..

[104] Yu P., Sun Y., Huang Z., Zhu F., Sun Y., Jiang L. (2020). The effects of ectomycorrhizal fungi on heavy metals’ transport in *pinus massoniana* and bacteria community in rhizosphere soil in mine tailing area. J. Hazard. Mater..

[105] Azuma T., Hayashi T. (2021). Disinfection of antibiotic-resistant bacteria in sewage and hospital effluent by ozonation. Ozone Sci. Eng..

[106] An X.L., Su J.Q., Li B., Ouyang W.Y., Zhao Y., Chen Q.L., Cui L., Chen H., Gillings M.R., Zhang T. (2018). Tracking antibiotic resistome during wastewater treatment using high throughput quantitative pcr. Environ. Int..

[107] Ogwugwa V.H., Oyetibo G.O., Amund O.O. (2021). Taxonomic profiling of bacteria and fungi in freshwater sewer receiving hospital wastewater. Environ. Res..

[108] Cheng J.H., Tang X.Y., Guan Z., Liu C. (2021). Occurrence of antibiotic resistome in farmland soils near phosphorus chemical industrial area. Sci. Total Environ..

[109] Cai C., Hui X., Yang W., Hua Y., Liu H., Dai X. (2022). Implications for mitigation of antibiotic resistance: Differential response of intracellular and extracellular antibiotic resistance genes to sludge fermentation coupled with thermal hydrolysis. Water Res..

[110] Wasserstein R.L., Lazar N.A. (2016). The ASA statement on *p*-values: Context, process, and purpose. Am. Stat..

[111] Agathokleous E. (2022). Environmental pollution impacts: Are *p* values over-valued?. Sci. Total Environ..

[112] Lee O.M., Kim H.Y., Park W., Kim T.H., Yu S. (2015). A comparative study of disinfection efficiency and regrowth control of microorganism in secondary wastewater effluent using UV, ozone, and ionizing irradiation process. J. Hazard. Mater..

[113] Ofori I., Maddila S., Lin J., Jonnalagadda S.B. (2018). Ozone initiated inactivation of *Escherichia coli* and *Staphylococcus aureus* in water: Influence of selected organic solvents prevalent in wastewaters. Chemosphere.

[114] Torii S., Itamochi M., Katayama H. (2020). Inactivation kinetics of waterborne virus by ozone determined by a continuous quench flow system. Water Res..

[115] Centers for Disease Control and Prevention (CDC) (2013). Guidelines for Environmental Infection Control in Health-Care Facilities Recommendations of CDC and the Healthcare Infection Control Practices Advisory Committee (HICPAC).

[116] Garcia A.B., Vinuela-Prieto J.M., Lopez-Gonzalez L., Candel F.J. (2017). Correlation between resistance mechanisms in *Staphylococcus aureus* and cell wall and septum thickening. Infect. Drug Resist..

[117] Azuma T., Hayashi T. (2021). On-site chlorination responsible for effective disinfection of wastewater from hospital. Sci. Total Environ..

[118] Choi Y., He H., Dodd M.C., Lee Y. (2021). Degradation kinetics of antibiotic resistance gene *mecA* of methicillin-resistant *Staphylococcus aureus* (MRSA) during water disinfection with chlorine, ozone, and ultraviolet light. Environ. Sci. Technol..

[119] Wang J., Bu L., Wu Y., Sun J., Li G., Zhou S. (2022). Disinfection profiles and mechanisms of *E. coli*, *S. aureus*, and *B. subtilis* in UV_365_/chlorine process: Inactivation, reactivation, and DBP formation. Sep. Purif. Technol..

[120] Torii S., David S.C., Larivé O., Cariti F., Kohn T. (2023). Observed kinetics of enterovirus inactivation by free chlorine are host cell-dependent. Environ. Sci. Technol..

[121] Qiao Z., Ye Y., Chang P.H., Thirunarayanan D., Wigginton K.R. (2018). Nucleic acid photolysis by UV254 and the impact of virus encapsidation. Environ. Sci. Technol..

[122] Liu X., Hu J.Y. (2020). Effect of DNA sizes and reactive oxygen species on degradation of sulphonamide resistance *sul*1 genes by combined UV/free chlorine processes. J. Hazard. Mater..

[123] He H., Choi Y., Wu S.J., Fang X., Anderson A.K., Liou S.Y., Roberts M.C., Lee Y., Dodd M.C. (2022). Application of nucleotide-based kinetic modeling approaches to predict antibiotic resistance gene degradation during UV- and chlorine-based wastewater disinfection processes: From bench- to full-scale. Environ. Sci. Technol..

[124] Ward C.P., Bowen J.C., Freeman D.H., Sharpless C.M. (2021). Rapid and reproducible characterization of the wavelength dependence of aquatic photochemical reactions using light-emitting diodes. Environ. Sci. Technol. Lett..

[125] Wang M., Ateia M., Awfa D., Yoshimura C. (2021). Regrowth of bacteria after light-based disinfection—What we know and where we go from here. Chemosphere.

[126] Lennon J.T., Muscarella M.E., Placella S.A., Lehmkuhl B.K., Zhou J. (2018). How, when, and where relic DNA affects microbial diversity. mBio.

[127] Burkert A., Douglas Thomas A., Waldrop Mark P., Mackelprang R., Atomi H. (2019). Changes in the active, dead, and dormant microbial community structure across a pleistocene permafrost chronosequence. Appl. Environ. Microbiol..

[128] Morrison C., Atkinson A., Zamyadi A., Kibuye F., McKie M., Hogard S., Mollica P., Jasim S., Wert E.C. (2021). Critical review and research needs of ozone applications related to virus inactivation: Potential implications for SARS-CoV-2. Ozone Sci. Eng..

[129] Epelle E.I., Macfarlane A., Cusack M., Burns A., Okolie J.A., Mackay W., Rateb M., Yaseen M. (2023). Ozone application in different industries: A review of recent developments. Chem. Eng. J..

[130] Loeb B.L. (2024). Ozone: A valuable tool for addressing today’s environmental issues. A review of forty-five years of Ozone: Science & Engineering. Ozone Sci. Eng..

[131] Wang R., Ji M., Zhai H., Guo Y., Liu Y. (2021). Occurrence of antibiotics and antibiotic resistance genes in wwtp effluent-receiving water bodies and reclaimed wastewater treatment plants. Sci. Total Environ..

[132] Wei Z., Feng K., Wang Z., Zhang Y., Yang M., Zhu Y.G., Virta M.P.J., Deng Y. (2021). High-throughput single-cell technology reveals the contribution of horizontal gene transfer to typical antibiotic resistance gene dissemination in wastewater treatment plants. Environ. Sci. Technol..

[133] Raza S., Shin H., Hur H.G., Unno T. (2022). Higher abundance of core antimicrobial resistant genes in effluent from wastewater treatment plants. Water Res..

[134] Huang Y.H., Liu Y., Du P.-P., Zeng L.J., Mo C.H., Li Y.W., Lü H., Cai Q.Y. (2019). Occurrence and distribution of antibiotics and antibiotic resistant genes in water and sediments of urban rivers with black-odor water in Guangzhou, South China. Sci. Total Environ..

[135] Cheng X., Xu J., Smith G., Zhang Y. (2021). Metagenomic insights into dissemination of antibiotic resistance across bacterial genera in wastewater treatment. Chemosphere.

[136] Jiang X., Liu L., Chen J., Fan X., Xie S., Huang J., Yu G. (2021). Antibiotic resistance genes and mobile genetic elements in a rural river in southeast China: Occurrence, seasonal variation and association with the antibiotics. Sci. Total Environ..

[137] Grenni P. (2022). Antimicrobial resistance in rivers: A review of the genes detected and new challenges. Environ. Toxicol. Chem..

[138] Stange C., Sidhu J.P.S., Toze S., Tiehm A. (2019). Comparative removal of antibiotic resistance genes during chlorination, ozonation, and UV treatment. Int. J. Hyg. Environ. Health.

[139] van Bruggen A.H.C., Goss E.M., Havelaar A., van Diepeningen A.D., Finckh M.R., Morris J.G. (2019). One health—Cycling of diverse microbial communities as a connecting force for soil, plant, animal, human and ecosystem health. Sci. Total Environ..

[140] Booton R.D., Meeyai A., Alhusein N., Buller H., Feil E., Lambert H., Mongkolsuk S., Pitchforth E., Reyher K.K., Sakcamduang W. (2021). One health drivers of antibacterial resistance: Quantifying the relative impacts of human, animal and environmental use and transmission. One Health.

[141] Auguet O., Pijuan M., Borrego C.M., Rodriguez-Mozaz S., Triadó-Margarit X., Giustina S.V.D., Gutierrez O. (2017). Sewers as potential reservoirs of antibiotic resistance. Sci. Total Environ..

[142] He P., Zhou Y., Shao L., Huang J., Yang Z., Lü F. (2019). The discrepant mobility of antibiotic resistant genes: Evidence from their spatial distribution in sewage sludge flocs. Sci. Total Environ..

[143] Zhang X., Li R. (2020). Variation and distribution of antibiotic resistance genes and their potential hosts in microbial electrolysis cells treating sewage sludge. Bioresour. Technol..

[144] Lee J., Jeon J.H., Shin J., Jang H.M., Kim S., Song M.S., Kim Y.M. (2017). Quantitative and qualitative changes in antibiotic resistance genes after passing through treatment processes in municipal wastewater treatment plants. Sci. Total Environ..

[145] Narciso-da-Rocha C., Rocha J., Vaz-Moreira I., Lira F., Tamames J., Henriques I., Martinez J.L., Manaia C.M. (2018). Bacterial lineages putatively associated with the dissemination of antibiotic resistance genes in a full-scale urban wastewater treatment plant. Environ. Int..

[146] Gwenzi W., Musiyiwa K., Mangori L. (2020). Sources, behaviour and health risks of antimicrobial resistance genes in wastewaters: A hotspot reservoir. J. Environ. Chem. Eng..

[147] World Health Organization (WHO) (2016). Quantitative Microbial Risk Assessment, Application for Water Safety Management.

[148] Le Page G., Gunnarsson L., Snape J., Tyler C.R. (2017). Integrating human and environmental health in antibiotic risk assessment: A critical analysis of protection goals, species sensitivity and antimicrobial resistance. Environ. Int..

[149] Pepper I.L., Brooks J.P., Gerba C.P. (2018). Antibiotic resistant bacteria in municipal wastes: Is there reason for concern?. Environ. Sci. Technol..

[150] Schages L., Wichern F., Kalscheuer R., Bockmühl D. (2020). Winter is coming—Impact of temperature on the variation of beta-lactamase and *mcr* genes in a wastewater treatment plant. Sci. Total Environ..

[151] Schoen M.E., Jahne M.A., Garland J., Ramirez L., Lopatkin A.J., Hamilton K.A. (2021). Quantitative microbial risk assessment of antimicrobial resistant and susceptible *Staphylococcus aureus* in reclaimed wastewaters. Environ. Sci. Technol..

[152] Wang L., Ye C., Guo L., Chen C., Kong X., Chen Y., Shu L., Wang P., Yu X., Fang J. (2021). Assessment of the UV/chlorine process in the disinfection of *Pseudomonas aeruginosa*: Efficiency and mechanism. Environ. Sci. Technol..

[153] Iakovides I.C., Manoli K., Karaolia P., Michael-Kordatou I., Manaia C.M., Fatta-Kassinos D. (2021). Reduction of antibiotic resistance determinants in urban wastewater by ozone: Emphasis on the impact of wastewater matrix towards the inactivation kinetics, toxicity and bacterial regrowth. J. Hazard. Mater..

[154] García-Espinoza J.D., Robles I., Durán-Moreno A., Godínez L.A. (2021). Photo-assisted electrochemical advanced oxidation processes for the disinfection of aqueous solutions: A review. Chemosphere.

[155] Li H., Dechesne A., He Z., Jensen M.M., Song H.L., Smets B.F. (2023). Electrochemical disinfection may increase the spread of antibiotic resistance genes by promoting conjugal plasmid transfer. Sci. Total Environ..

[156] Kiejza D., Kotowska U., Polińska W., Karpińska J. (2021). Peracids—New oxidants in advanced oxidation processes: The use of peracetic acid, peroxymonosulfate, and persulfate salts in the removal of organic micropollutants of emerging concern—A review. Sci. Total Environ..

[157] Sahulka S.Q., Bhattarai B., Bhattacharjee A.S., Tanner W., Mahar R.B., Goel R. (2021). Differences in chlorine and peracetic acid disinfection kinetics of *Enterococcus faecalis* and *Escherichia fergusonii* and their susceptible strains based on gene expressions and genomics. Water Res..

[158] Luo L.W., Wu Y.H., Chen G.Q., Wang H.B., Wang Y.H., Tong X., Bai Y., Xu Y.Q., Zhang Z.-W., Ikuno N. (2022). Chlorine-resistant bacteria (CRB) in the reverse osmosis system for wastewater reclamation: Isolation, identification and membrane fouling mechanisms. Water Res..

[159] Wang R., Alamin M., Tsuji S., Hara-Yamamura H., Hata A., Zhao B., Ihara M., Honda R. (2022). Removal performance of SARS-CoV-2 in wastewater treatment by membrane bioreactor, anaerobic-anoxic-oxic, and conventional activated sludge processes. Sci. Total Environ..

[160] Zhou Z., Tran P.Q., Kieft K., Anantharaman K. (2020). Genome diversification in globally distributed novel marine proteobacteria is linked to environmental adaptation. ISME J..

[161] Niestępski S., Harnisz M., Ciesielski S., Korzeniewska E., Osińska A. (2020). Environmental fate of *Bacteroidetes*, with particular emphasis on *Bacteroides fragilis* group bacteria and their specific antibiotic resistance genes, in activated sludge wastewater treatment plants. J. Hazard. Mater..

[162] Wallace M.J., Jean S., Wallace M.A., Burnham C.A.D., Dantas G. (2022). Comparative genomics of *Bacteroides fragilis* group isolates reveals species-dependent resistance mechanisms and validates clinical tools for resistance prediction. mBio.

[163] Alexander J., Knopp G., Dötsch A., Wieland A., Schwartz T. (2016). Ozone treatment of conditioned wastewater selects antibiotic resistance genes, opportunistic bacteria, and induce strong population shifts. Sci. Total Environ..

[164] Czekalski N., Imminger S., Salhi E., Veljkovic M., Kleffel K., Drissner D., Hammes F., Bürgmann H., von Gunten U. (2016). Inactivation of antibiotic resistant bacteria and resistance genes by ozone: From laboratory experiments to full-scale wastewater treatment. Environ. Sci. Technol..

[165] Gomi R., Matsuda T., Yamamoto M., Chou P.H., Tanaka M., Ichiyama S., Yoneda M., Matsumura Y. (2018). Characteristics of carbapenemase-producing *Enterobacteriaceae* in wastewater revealed by genomic analysis. Antimicrob. Age. Chemother..

[166] Sekizuka T., Yatsu K., Inamine Y., Segawa T., Nishio M., Kishi N., Kuroda M. (2018). Complete genome sequence of a *bla*_kpc-2_-positive *Klebsiella pneumoniae* strain isolated from the effluent of an urban sewage treatment plant in Japan. mSphere.

[167] Sekizuka T., Inamine Y., Segawa T., Kuroda M. (2019). Characterization of NDM-5- and CTX-M-55-coproducing *Escherichia coli* GSH8M-2 isolated from the effluent of a wastewater treatment plant in Tokyo Bay. Infect. Drug Resist..

[168] Teixeira P., Tacão M., Pureza L., Gonçalves J., Silva A., Cruz-Schneider M.P., Henriques I. (2020). Occurrence of carbapenemase-producing *Enterobacteriaceae* in a portuguese river: *bla*_NDM_, *bla*_KPC_ and *bla*_GES_ among the detected genes. Environ. Pollut..

[169] Hiller C.X., Hübner U., Fajnorova S., Schwartz T., Drewes J.E. (2019). Antibiotic microbial resistance (AMR) removal efficiencies by conventional and advanced wastewater treatment processes: A review. Sci. Total Environ..

[170] Pazda M., Kumirska J., Stepnowski P., Mulkiewicz E. (2019). Antibiotic resistance genes identified in wastewater treatment plant systems –A review. Sci. Total Environ..

[171] Piña B., Bayona J.M., Christou A., Fatta-Kassinos D., Guillon E., Lambropoulou D., Michael C., Polesel F., Sayen S. (2020). On the contribution of reclaimed wastewater irrigation to the potential exposure of humans to antibiotics, antibiotic resistant bacteria and antibiotic resistance genes—NEREUS COST Action ES1403 position paper. J. Environ. Chem. Eng..

