# Supplementary material for: On-Site Inactivation for Disinfection of Antibiotic-Resistant Bacteria in Hospital Effluent by UV and UV-LED
Source: Antibiotics (Basel). 2024 Jul 29;13(8):711. doi: 10.3390/antibiotics13080711 (PMC11350808; doi:10.3390/antibiotics13080711)
Supplement: Supplementary file 1 [file antibiotics-13-00711-s001.zip › antibiotics-3111016-supplementary.pdf]

Supplemental Information for

On-Site Inactivation for Disinfection of Antibiotic-Resistant Bacteria in Hospital Effluent by UV and UV-LED

Takashi Azuma [1,*], Masaru Usui [2], Tomohiro Hasei [1] and Tetsuya Hayashi [1]

Affiliation:

1 Department of Pharmacy, Osaka Medical and Pharmaceutical University, Takatsuki 569-1094, Japan;

tomohiro.hasei@ompu.ac.jp (T.H.); tetsuhaya456@gmail.com (T.H.)

2 Food Microbiology and Food Safety, Department of Health and Environmental Sciences, School of

Veterinary Medicine, Rakuno Gakuen University, Ebetsu 069-8501, Japan; usuima@rakuno.ac.jp

* Correspondence: takashi.azuma@ompu.ac.jp; Tel./Fax: +81-72-690-1055

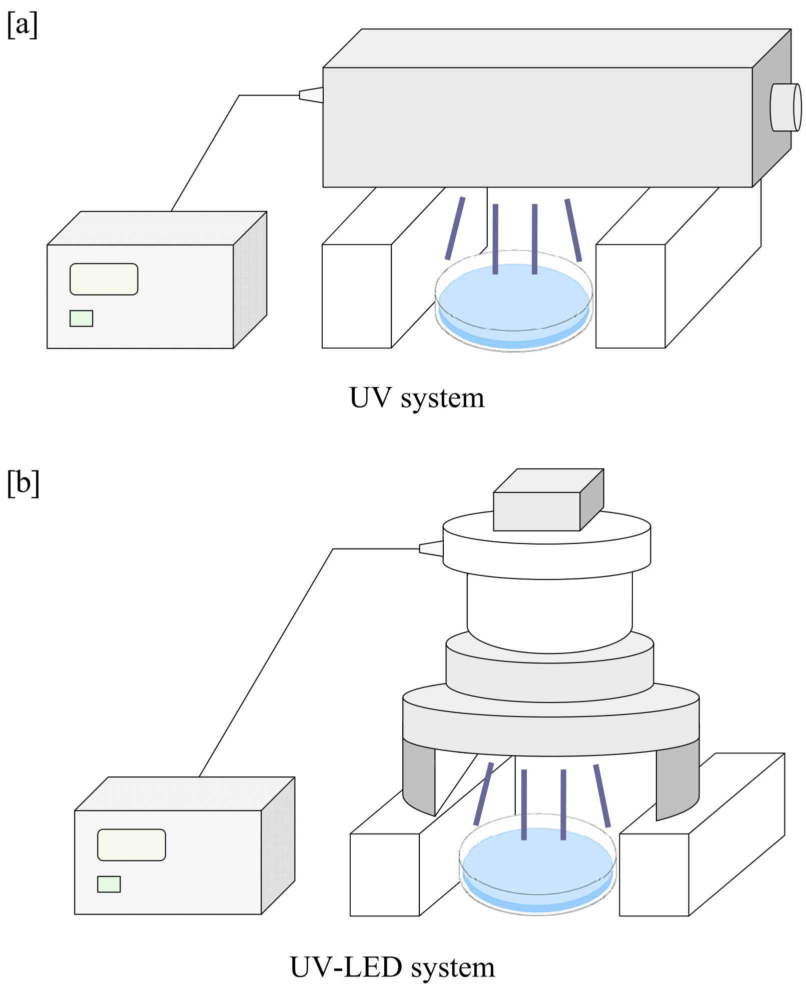

Figure S1. UV inactivation reactor used for the evaluation. ([a]: UV system, [b]: UV-LED system)

Table S1. Primers and PCR conditions for AMRGs analyses [1-4]

Gene Source	Sequence (5'–3')	Amplification Length (bp)	Annealing temperature (°C)	Linearity (r^2)	Efficiency (%)
bla $_{IMP}$ [1]	FW GGAATAGAGTGGCTTAAYTCTC<br>RV GGTTTAAYAAAACAACCACC	233	57	0.99	95
bla $_{TEM}$ [2]	FW GCKGCCAACTTACTTCTGACAACG<br>RV CTTTATCCGCCTCCATCCAGTCTA	55	247	0.99	115
bla $_{CTX\text{-}M}$ [3]	FW ATTCCRGGCGAYCCGCGTGATACC<br>RV ACCGCGATATCGTTGGTGGTGCCAT	62	227	0.99	112
16S rRNA [4]	FW CCTACGGGAGGCAGCAG<br>RV ATTACCGCGGCTGCTGG	192	60	0.99	98

Table S2. Half-life of each antimicrobial during UV and UV-LED treatment of WWTP wastewater and hospital effluent.

Bacteria	Half-life (min)			
	WWTP wastewater		Hospital effluent	
	UV (254 nm)	UV-LED (285 nm)	UV (254 nm)	UV-LED (285 nm)
CRE	0.1	0.3	0.1	0.1
ESBL-E	0.1	0.2	0.1	0.5
MDRA	0.1	0.1	0.5	0.5
MDRP	0.2	0.5	0.2	0.5
MRSA	0.2	0.4	0.4	0.9
VRE	0.1	0.1	0.2	0.2
Acinetobacter	0.1	0.1	0.3	0.5
Enterococcus	0.1	0.3	0.1	0.2
Escherichia coli	0.1	0.2	0.1	0.3
Pseudomonas aeruginosa	0.1	0.1	0.1	0.4
Staphylococcus aureus	0.3	0.4	0.3	0.5

The values for the WWTP wastewater model were prepared by mixing the WWTP influent and secondary effluent (1:9 [v/v]. CRE, carbapenem-resistant *Enterobacteriaceae*; ESBL-E, extended-spectrum *β*-lactamase-producing *Enterobacteriaceae*; MDRA, multidrug-resistant *Acinetobacter*; MDRP, multidrug-resistant *Pseudomonas aeruginosa*; MRSA, methicillin-resistant *Staphylococcus aureus*; VRE, vancomycin-resistant *Enterococcus*).

Table S3. Occurrence of AMRGs in WWTP wastewater and hospital effluent.

Gene Source	Log copy/mL			
	WWTP wastewater		Hospital effluent	
	Mean	SD	Mean	SD
bla $_{IMP}$	4.5	4.0	3.3	2.3
bla $_{TEM}$	2.7	1.9	5.9	5.4
bla $_{CTX-M}$	1.9	1.8	2.9	1.8
16S rRNA	5.0	3.8	5.7	4.9
